# Functional Substitution by TAT-Utrophin in Dystrophin-Deficient Mice

**DOI:** 10.1371/journal.pmed.1000083

**Published:** 2009-05-26

**Authors:** Kevin J. Sonnemann, Hanke Heun-Johnson, Amy J. Turner, Kristen A. Baltgalvis, Dawn A. Lowe, James M. Ervasti

**Affiliations:** 1 Department of Biochemistry, Molecular Biology and Biophysics, University of Minnesota, Minneapolis, Minnesota, United States of America; 2 Program in Physical Therapy, University of Minnesota, Minneapolis, Minnesota, United States of America; University of Oxford, United Kingdom

## Abstract

**Background:**

The loss of dystrophin compromises muscle cell membrane stability and causes Duchenne muscular dystrophy and/or various forms of cardiomyopathy. Increased expression of the dystrophin homolog utrophin by gene delivery or pharmacologic up-regulation has been demonstrated to restore membrane integrity and improve the phenotype in the dystrophin-deficient *mdx* mouse. However, the lack of a viable therapy in humans predicates the need to explore alternative methods to combat dystrophin deficiency. We investigated whether systemic administration of recombinant full-length utrophin (Utr) or ΔR4-21 “micro” utrophin (μUtr) protein modified with the cell-penetrating TAT protein transduction domain could attenuate the phenotype of *mdx* mice.

**Methods and Findings:**

Recombinant TAT-Utr and TAT-μUtr proteins were expressed using the baculovirus system and purified using FLAG-affinity chromatography. Age-matched *mdx* mice received six twice-weekly intraperitoneal injections of either recombinant protein or PBS. Three days after the final injection, mice were analyzed for several phenotypic parameters of dystrophin deficiency. Injected TAT-μUtr transduced all tissues examined, integrated with members of the dystrophin complex, reduced serum levels of creatine kinase (11,290±920 U versus 5,950±1,120 U; PBS versus TAT), the prevalence of muscle degeneration/regeneration (54%±5% versus 37%±4% of centrally nucleated fibers; PBS versus TAT), the susceptibility to eccentric contraction-induced force drop (72%±5% versus 40%±8% drop; PBS versus TAT), and increased specific force production (9.7±1.1 N/cm^2^ versus 12.8±0.9 N/cm^2^; PBS versus TAT).

**Conclusions:**

These results are, to our knowledge, the first to establish the efficacy and feasibility of TAT-utrophin-based constructs as a novel direct protein-replacement therapy for the treatment of skeletal and cardiac muscle diseases caused by loss of dystrophin.

## Abstract 2

James Ervasti and colleagues show that injection of a truncated form of utrophin transduced all tissues examined, integrated with members of the dystrophin complex, and reduced serum levels of creatine kinase in a mouse model of muscular dystrophy.

## Editors' Summary

**Background:**

Muscular dystrophies are genetic (inherited) diseases in which the body's muscles gradually weaken and degenerate. The commonest and most severe muscular dystrophy—Duchenne muscular dystrophy—affects 1 in 3,500 boys (girls can be carriers of the disease but rarely have any symptoms). At birth, these boys seem normal but the symptoms of their disease begin to appear in early childhood. Affected children may initially have difficulty walking or find it to hard to sit or stand independently. As they age, their muscle strength progressively declines and most affected boys are confined to a wheelchair by the time they are 12 years old. The muscles involved in breathing also weaken and the heart muscle becomes enlarged. Few boys with Duchenne muscular dystrophy live beyond their early 20 s, usually dying from breathing or heart problems. At present there is no cure for Duchenne muscular dystrophy. However, physical therapy and treatment with steroids can prolong the ability of patients to walk, and assisted ventilation can help with their breathing.

**Why Was This Study Done?:**

In all muscular dystrophies, one of the proteins needed to build and maintain healthy muscles is missing or nonfunctional because of a genetic change (mutation). In Duchenne muscular dystrophy the mutation is in dystrophin, a protein that is involved in the formation of the dystrophin–glycoprotein complex. This complex normally sits in the membranes that surround muscle fibers and protects these membranes from damage during muscle contraction. Consequently, in Duchenne muscular dystrophy, the muscle fiber membranes become damaged and eventually the muscle fibers die. Thus, if functional dystrophin could be introduced into the muscles of patients with Duchenne muscular dystrophy, it might be possible to reduce their symptoms and prolong their lives. Indeed, the effects of dystrophin deficiency in the dystrophin-deficient *mdx* mouse can be reduced by the introduction of an artificial gene that expresses dystrophin or the closely related protein utrophin. Unfortunately, this gene therapy approach has not yet been effectively demonstrated in humans. In this study, therefore, the researchers investigate whether utrophin protein can be introduced directly into dystrophin-deficient mouse muscles by exposing the muscle cells to utrophin fused to the protein transduction domain of the HIV-1 TAT protein. Most proteins will not cross cell membranes, but proteins fused to this cell-penetrating domain readily enter many cell types, including muscle cells.

**What Did the Researchers Do and Find?:**

The researchers injected full-length utrophin fused to the TAT protein transduction domain (TAT-Utr) and a short, “micro” version of utrophin fused to the same domain (TAT-μUtr) into the abdomens of *mdx* mice and looked to see where the proteins ended up. After two injections, both proteins were present in a wide range of tissues and organs, including several types of muscle. However, the levels of TAT-Utr were much lower than those of TAT-μUtr. Next, the researchers injected another group of *mdx* mice with TAT-μUtr six times over three weeks. Again, TAT-μUtr was present in all the tissues that the researchers examined. Furthermore, μUtr–glycoprotein complexes formed in the TAT-μUtr injected *mdx* mice and the membrane integrity and overall health of the dystrophin-deficient muscles of the *mdx* mice improved compared to *mdx* mice treated with saline. Finally, the researchers report, TAT-μUtr injections greatly improved the contractile performance of the muscles of the *mdx* mice.

**What Do These Findings Mean?:**

These findings provide the first demonstration that injection of TAT-utrophin protein fusions may provide a way to treat muscular dystrophies caused by the loss of dystrophin. However, although this direct protein-replacement therapy looks hopeful, approaches that work in animals do not necessarily work in people. In particular, for this approach to work in patients with muscular dystrophy, it would be necessary to give frequent, high-dose injections of the TAT-μUtr fusion protein, a process that could eventually trigger a deleterious immune response. Nevertheless, the researchers suggest that by combining this novel approach with other approaches that also increase utrophin expression, it might be possible to prevent or delay the development of the symptoms of Duchenne muscular dystrophy.

**Additional Information:**

Please access these Web sites via the online version of this summary at http://dx.doi.org/10.1371/journal.pmed.1000085.

- The US National Institute of Neurological Disorders and Stroke provides information on muscular dystrophy and ongoing research into possible treatments (in English and Spanish)
- The US National Human Genome Research Institute also provides basic information on Duchenne muscular dystrophy and links to additional resources
- The UK National Health Service Choices Web site has pages for patients and caregivers on muscular dystrophy
- The Nemours Foundation provides information about muscular dystrophy for parents, children, and teenagers
- For links to further resources on muscular dystrophy, see also MedlinePlus

## Introduction

Duchenne muscular dystrophy (DMD) is the most prevalent form of human muscular dystrophy and is caused by mutations in dystrophin, a 427 kDa cytoskeletal protein necessary for proper membrane stability in muscle [1]. Although recent advances in cell-, gene-, oligonucleotide-, and small molecule-based therapies have identified several promising approaches to counteract the effects of dystrophin deficiency in animal models [2]–[9], there are currently no effective therapies for humans with DMD. We have chosen to pursue the therapeutic potential of utrophin (Utr), an autosomal homolog of dystrophin that corrects all known phenotypes of the dystrophin-deficient *mdx* mouse when transgenically overexpressed to sufficient levels [10]. Our ability to express and purify scalable quantities of recombinant full-length and truncated utrophins [11], combined with the availability of cell-penetrating peptides, provides an attractive potential method for directly boosting utrophin levels in vivo.

The protein transduction domain (PTD) of the HIV-1 TAT protein has been used to effect cellular entry of TAT-fusion proteins and oligonucleotides into every tissue type examined, including muscle cells [9],[12],[13]. However, the “carrying capacity” of the TAT PTD is unknown, and the molecular weight of full-length utrophin is over three times larger than the largest TAT-fusion protein described to date [12]. Therefore, we chose to compare a smaller truncated utrophin construct with full-length utrophin. Although protein truncation potentially compromises utrophin function [14], Odom et al. recently demonstrated that a virus-delivered truncated “micro” utrophin construct extended the lifespan and significantly improved the phenotype of *mdx*/*utrn* ^−/−^ mice [15]. Therefore, we sought to determine whether TAT-mediated full-length or micro-utrophin delivery is a viable therapeutic option for the treatment of dystrophinopathy. We expressed chimeric proteins encoding the TAT PTD fused to both full-length Utr (TAT-Utr) and ΔR4–21 “micro” Utr (TAT-μUtr) and then assessed the ability of purified full-length TAT-Utr and TAT-μUtr to prevent the dystrophic phenotype of the *mdx* mouse.

## Methods

### TAT-Utr and TAT-μUtr

The 5′ coding sequence of a previously described 16 kb murine utrophin baculoviral expression construct [11] was subcloned into the plasmid pTAT (a gift from Steven Dowdy, UCSD). A Kozak consensus sequence and FLAG-epitope were engineered in-frame at the extreme 5′ end of TAT-Utr before reinsertion of the modified fragment into the baculovirus expression construct. Protein expression and purification was carried out as described [11]. The construction of TAT-μUtr is detailed in Text S1. The purified proteins were sterilized for injection by passage through a 0.22 µm filter and injected into the intraperitoneal (IP) cavity of *mdx* mice at a concentration of 1.0 to 3.0 mg/ml.

### Protein Labeling

1 mg of purified full-length TAT-Utr or TAT-μUtr was diluted to 1.0 mg/ml in PBS and labeled with IRDye 800CW - High MW Protein Labeling Kit (Li-Cor Biosciences) according to the manufacturer's instructions. The dye is stably coupled to free amines through an NHS ester reactive group. Unincorporated dye was separated by passage through a desalting spin column and the protein concentration of the labeled protein was determined. The labeled proteins were filter-sterilized prior to injection as above.

### Infrared Imaging

All tissue/organ and in vivo scanning was performed on freshly killed or anesthetized mice using the Odyssey Infrared Imaging System (Li-Cor Biosciences) using both the 700 and 800 nm channels.

For tissue transduction studies, five *mdx* mice received two injections each, 72 h apart, of either labeled full-length TAT-Utr (20 µg/g body weight), TAT-μUtr (8.5 µg/g body weight), or equal volume injections of sterile PBS. Mice were humanely killed 72 h after the second injection and their tissues/organs were immediately dissected and scanned before freezing for subsequent protein analysis. Fluorescence intensity data from the 800 nm channel (labeled protein) was normalized first to the 700 nm channel (background) of the same tissue/organ before normalization to PBS tissue/organ.

For decay time course experiments, four mice were singly injected with equimolar amounts of either full-length TAT-Utr (two groups of four mice) or TAT-μUtr (two groups of four mice). At 3, 24, 48, and 72 h postinjection, all living mice were scanned and one mouse from each group was killed for tissue protein analyses.

### Treatment

C57Bl/10ScSn-*Dmd^mdx^*/J (The Jackson Laboratory, Bar Harbor, ME) littermates were treated in parallel (unbiased by gender), and received a dose of either 5 (0.25×; *n* = 2), 10 (0.5×; *n* = 3), 20 (1×; *n* = 5), 40 (2×; *n* = 3), or 100 µg (5×; *n* = 3) of full-length TAT-Utr/g body weight or 8.5 µg TAT-μUtr/g body weight (*n* = 8), while *mdx* littermate mice received equal volume injections of sterile PBS (*n* = 13). A total of six twice-weekly injections were administered over 3 wk, beginning at 18 d and culminating at 35 d of age. At 38 d of age, serum and tissue were collected for creatine kinase, Western blot, immunofluorescence, histological, and contractile analyses. For data analyses, PBS-injected animals were randomly assigned to two groups; one group (*n* = 7) was used for comparison to full-length TAT-Utr-treated animals while the other group (*n* = 6) was used for comparison to TAT-μUtr-treated animals. Animals were housed and treated in accordance with the standards set by the University of Minnesota Institutional Animal Care and Use Committee.

### Protein Extracts

Tissues were dissected from freshly killed mice and snap-frozen in liquid nitrogen before subsequent analyses. For SDS-extracts, frozen tissue was pulverized and the protein extracted as previously described [11]. For samples enriched in membrane glycoproteins using wheat germ agglutinin (WGA) affinity chromatography, pulverized muscle was instead solubilized 1∶10 (w∶v) in 5% digitonin solubilization buffer for 1 h at 4°C. The solubilate was centrifuged at 1,000 rpm for 10 min and the supernatant then loaded onto equilibrated WGA beads (50 µl of beads per 1 ml of supernatant) and mixed end-over-end overnight at 4°C. Beads were then pelleted and washed three times in 10% digitonin wash buffer before protein was eluted in 0.3 M NAG elution buffer.

### Electrophoresis/Western Blotting

For size separation, 4%–16% BN gels were loaded with 100 µg of WGA-eluted protein from TAT-μUtr-treated *mdx*, PBS-injected *mdx*, and PBS-injected *mdx* spiked with 0.1 µg of fluorescently labeled TAT-μUtr. The gels were run for approximately 2 h at 70 V, and then increased to 150 V for 1 h. Once the dye front had migrated halfway through the gel the cathode buffer was changed from a Coomassie G250-containing cathode buffer to a dyeless cathode buffer. After the dye front had sufficiently migrated through the native gel, the appropriate lanes were excised and loaded on top of a conventional 3%–12% gradient SDS-PAGE gel.

All Western blotting was performed as described [11] using the following primary antibodies: utrophin mAb 8A4 (1∶50; Santa Cruz), anti-β-dystroglycan mAb NCL-b-DG (1∶50; Novocastra), anti-α-sarcoglycan mAb NCL-a-Sarc (1∶50; Novocastra), anti-γ-sarcoglycan mAb NCL-g-Sarc (1∶50; Novocastra), anti-dystrobrevin (1∶50; Novocastra), anti-nNOS pAb Z-RNN3 (1∶1000; Invitrogen), anti-actin mAbs C4 and AC-15 (1∶1000; Sigma), and anti-FLAG mAb M2 (1∶1000; Sigma) and pAb ANTI-FLAG (1∶1000; Sigma). Secondary antibodies were diluted (1∶5000) and detected with the Odyssey Infrared Imaging System (Li-Cor Biosciences) using the 700 and 800 nm channels.

### Histological and Morphometric Analysis

Individual muscles were dissected, coated with OCT (TissueTek), and rapidly frozen in liquid nitrogen-cooled isopentane. Cryosections of 10 µm thickness were cut on a Leica CM3050 cryostat and stained with hematoxylin and eosin–phloxine. Images were collected on a Zeiss Axiovert 25 microscope and compiled into montages of entire sections in ImagePro Plus and exported to Scion Image for morphometric analyses. The percentage of centrally nucleated fibers (CNFs) and fiber diameters were determined from one muscle of each mouse, with every fiber scored for CNF analysis and ∼700 fiber diameters measured per muscle section. A Student's *t* test was used to compare both average CNF values and average fiber diameter.

### Immunofluorescence

Cryosections of 10 µm thickness were stained with primary antibodies as previously described [16]. Confocal images were obtained using an inverted Olympus Fluoview 1000 confocal microscope at the Biomedical Image Processing Lab and imported into CorelDraw 10 for figure preparation. Primary monoclonal antibodies used were identical to those described for Western blotting above.

### Contractile Properties

All contractile assays are detailed in Text S1.

### Serum CK Analysis

Retro-orbital bleeds were performed on anesthetized mice as previously described [16]. Data were collected in U/ml and compared by Student's *t* test.

## Results

### Expression and Characterization of Recombinant TAT-Fusion Proteins

**Figure 1:**
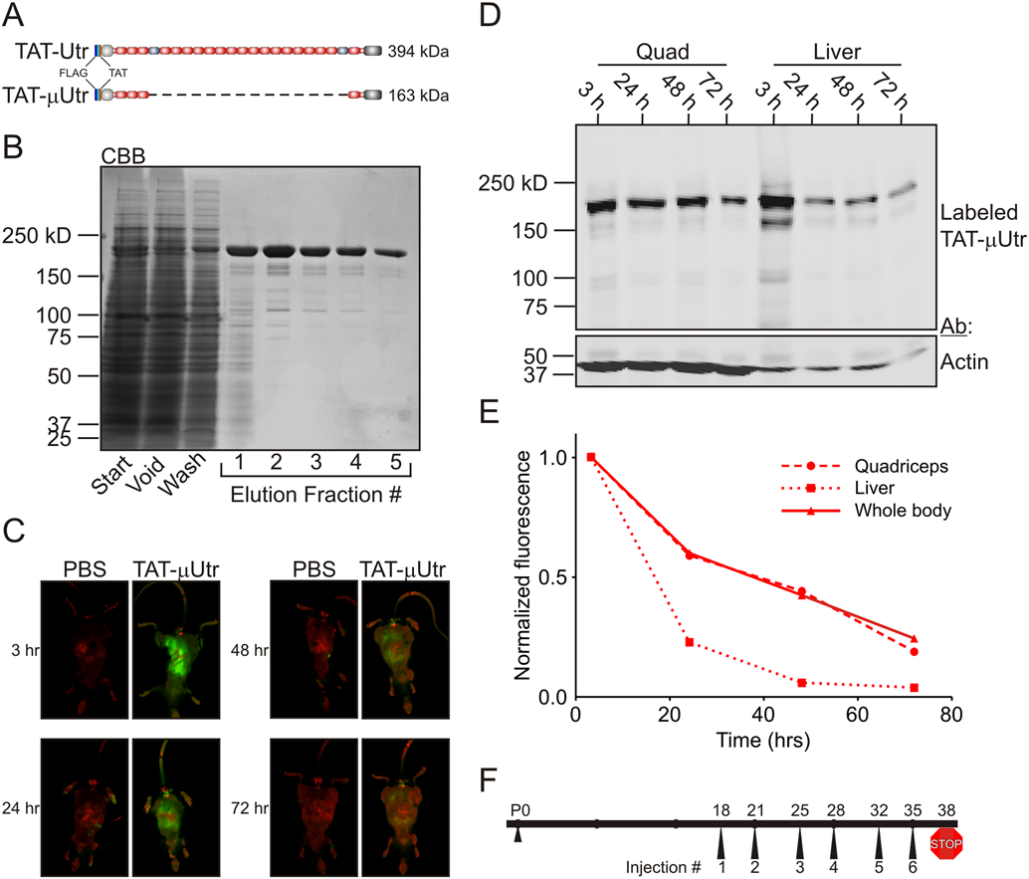
TAT-μUtr stability in vivo. (A) Schematic of TAT-Utr and TAT-μUtr with the orientation of the N-terminal FLAG and TAT epitopes noted. TAT-μUtr is deleted for spectrin-like repeats 4–21 (dashed line) of the utrophin middle rod domain but retains all N- and C-terminal domains. (B) Coomassie blue-stained gel of a typical TAT-μUtr purification from *Sf*9 insect cell lysate using anti-FLAG M2 affinity chromatography. Typical protein yield was ∼1.5 mg of purified protein per gram of *Sf*9 cell paste. (C) Infrared in vivo scanning of *mdx* littermate mice after a single IP injection of fluorescently labeled TAT-μUtr. Mice were scanned 3, 24, 48, and 72 h postinjection with one mouse killed at each time point for tissue analysis. Green fluorescence corresponds to TAT-μUtr while red signal is tissue autofluorescence. (D) Infrared scan of a nitrocellulose membrane after SDS-PAGE transfer of quadriceps and liver SDS protein extracts from mice described in (C). The same membrane was Western blotted for actin as a loading control. (E) Quantification of the TAT-μUtr fluorescence decay in whole-body (C) and tissue extracts (D) over time. Whole body fluorescence was normalized to body autofluorescence (red signal), while Western blot/tissue extract fluorescence was normalized to protein load on SDS-PAGE gels stained with Coomassie blue after transfer. (F) Treatment protocol schematic for administering TAT-μUtr to *mdx* mice. Each treated mouse received six twice-weekly IP injections between 18 and 35 d of age and was then humanely killed at 38 d of age.

Full-length TAT-Utr and TAT-μUtr were expressed in baculovirus-infected *Sf*9 cells and purified using anti-FLAG affinity chromatography (Figures 1A and S1A). As expected from biochemical studies that have mapped the actin-binding interface of utrophin [14], deletion of spectrin-like repeats 4–21 of the middle rod domain resulted in a 5- to 10-fold reduction in actin binding affinity for TAT-μUtr (Figure S1B). However, the measured affinity of TAT-μUtr for actin was within the range of affinities reported for truncated dystrophin constructs that ameliorated the dystrophic phenotype in vivo [17],[18].

### TAT Protein Transduction and Stability

In order to assess the cellular transduction of TAT-μUtr and full-length TAT-Utr in vivo, we labeled each protein with an infrared-excitable fluorescent dye and administered two injections of labeled protein at equimolar concentrations (TAT-μUtr, 8.5 µg/g body weight; full-length TAT-Utr, 20 µg/g body weight) 72 h apart to *mdx* littermate mice. Mice were killed 72 h after the second injection and their tissues/organs prepared for fluorescence and protein analyses. Both infrared scanning and Western blot analysis using a FLAG epitope-specific antibody identified TAT-μUtr in every tissue/organ examined from treated mice (Figure S2A) while no TAT-μUtr was observed in sham PBS-injected *mdx* littermates. Infrared fluorescence scanning of tissues from mice injected with labeled protein revealed widespread uptake of both TAT-μUtr and full-length TAT-Utr when examined in cross-section (Figure S2B) and at the macroscopic level in all tissues analyzed (Figure S2C and S2D). However, the extent of transduction by TAT-μUtr and full-length TAT-Utr was tissue-dependent as the fluorescence intensity of different tissues and organs varied dramatically (Figure S2E). While some differences may be explained by the proximity of the tissue/organ to the injection site (diaphragm and several peritoneal organs showed high levels of fluorescence), it is unclear why other tissues/organs were preferentially transduced while neighboring tissues/organs were not (e.g., quadriceps versus gastrocnemius, lungs versus heart). More importantly, full-length TAT-Utr levels appeared lower than TAT-μUTR as determined by both tissue fluorescence intensity (Figure S2E) and Western blot analysis (Figure S2F), suggesting that TAT-μUTR either transduced tissues more effectively or was more stable in vivo.

To assess the stability of injected TAT-utrophin proteins in vivo, we administered a single injection of fluorescently tagged TAT-μUtr or full-length TAT-Utr (TAT-μUtr, 8.5 µg/g body weight; full-length TAT-Utr, 20 µg/g body weight) to *mdx* littermate mice and measured whole-body fluorescence intensity at 3, 24, 48, and 72 h postinjection (Figures 1C and S3A). At each time point one animal was killed and its tissues analyzed by SDS-PAGE/Western blot to determine whether fluorescence in tissue extracts was consistent with whole-body fluorescence (Figure 1D). Although TAT-μUtr levels decayed most rapidly in liver (Figure 1C and 1D), the fluorescence decay in both liver and quadriceps extracts closely paralleled the whole-body fluorescence decay and approached baseline levels 72 h postinjection (Figure 1E). Therefore, whole-body fluorescence served as a reliable readout for TAT-μUtr protein levels in vivo in subsequent stability assays. Because similar whole-body fluorescence decay profiles were observed for TAT-μUtr and full-length TAT-Utr (Figures 1E and S3B), we concluded that the higher levels of labeled protein detected in mice injected with TAT-μUtr were due to the greater transduction efficiency of the truncated protein.

**Figure 2:**
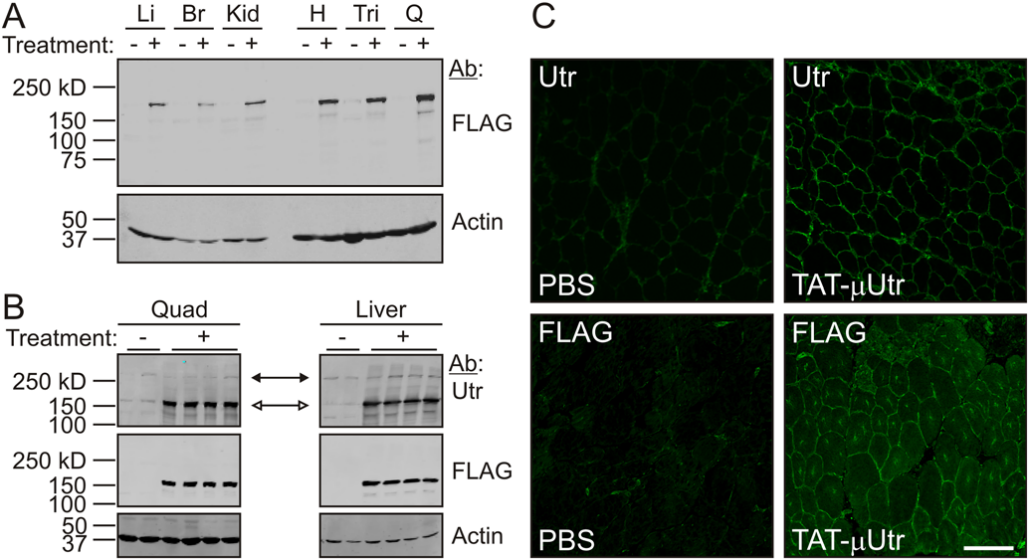
TAT-μUtr transduces *mdx* tissue. (A) Western blot analysis of liver (Li), brain (Br), kidney (Kid), heart (H), triceps (Tri), and quadriceps (Q) SDS protein extracts from 38-d-old mice after six injections of PBS (−) or TAT-μUtr (+). FLAG immunoreactivity corresponding to TAT-μUtr was observed in all treated tissues. The same blot was probed for actin as a loading control. (B) Western blots of quadriceps and liver SDS extracts probed with an anti-utrophin antibody demonstrated TAT-μUtr (open arrows) had no effect on endogenous utrophin levels (solid arrows). The same blots were also probed for FLAG and actin as a loading control. (C) Immunofluorescence analysis of 10 µm thick muscle cryosections from PBS- or TAT-μUtr-treated mice using anti-utrophin and anti-FLAG antibodies. Scale bar = 100 µm.

The 5-fold higher molar yield of TAT-μUtr after expression and purification (unpublished data) combined with its enhanced transduction efficiency (Figures 1 and 2) led us to focus on the therapeutic potential of TAT-μUtr. We administered twice-weekly injections based on the discovery that a fraction of injected protein remained stable for at least three days postinjection (Figure 1D). The time frame of our injection protocol was influenced by (1) the amount of transgenic utrophin expression required to improve the *mdx* phenotype [10],[11] and (2) evidence suggesting that utrophin up-regulation prior to the onset of muscle degeneration/regeneration in the *mdx* mouse (∼21 d of age) was most effective in alleviating the dystrophic phenotype [19]. Thus, *mdx* mice received six twice-weekly IP injections of TAT-μUtr (8.5 µg/g body weight) into *mdx* mice beginning at day 18 postpartum (Figure 1F). Treated mice were killed 3 d after the sixth injection, transcardially perfused with excess PBS to clear residual injected protein from the vasculature, and assessed for several parameters of the dystrophic phenotype.

### TAT-μUtr Forms a μUGC

A total of eight *mdx* mice (each randomly selected without bias for gender at the time of the first injection) received the six-injection protocol while *mdx* littermates were injected with equal volumes of sterile PBS. To assess whether the injected TAT-μUtr achieved systemic transduction, we first analyzed several tissue extracts by Western blot and observed strong FLAG immunoreactivity corresponding to TAT-μUtr only in treated samples (Figure 2A). The relative levels of TAT-μUtr between tissues in mice receiving six injections was greater than in mice receiving two injections, and striated muscle extracts exhibited stronger TAT-μUtr immunoreactivity after six injections compared to nonmuscle tissues (compare Figures 2A and S2A). Although the mechanism leading to this finding is unknown, it is perhaps due to differences in the inherent stability of TAT-μUtr in muscle versus nonmuscle tissues (Figure 1D and 1E). Importantly, Western blotting with a utrophin-specific antibody showed that the amount of endogenous full-length utrophin was unchanged in tissues from mice injected with TAT-μUtr (Figure 2B), indicating that any effect of TAT-μUtr on the dystrophic phenotype was not due to alterations in endogenous utrophin expression. To assess the intracellular localization of TAT-μUtr, we stained quadriceps muscle cryosections with primary antibodies to either utrophin or the FLAG epitope and observed intense staining along the periphery of muscle cells in treated mice, but only limited staining with a utrophin-specific antibody in PBS-injected mice (Figure 2C). The staining pattern for TAT-μUtr was consistent with the location of dystrophin in wild-type muscle cells [1] and transgenically overexpressed utrophin in *mdx* tissue [10], indicating that most internalized TAT-μUtr was appropriately targeted to the subsarcolemmal space. Taken together, the Western blot and immunofluorescence data in Figure 2 demonstrate both stable systemic transduction and subcellular localization of TAT-μUtr.

**Figure 3:**
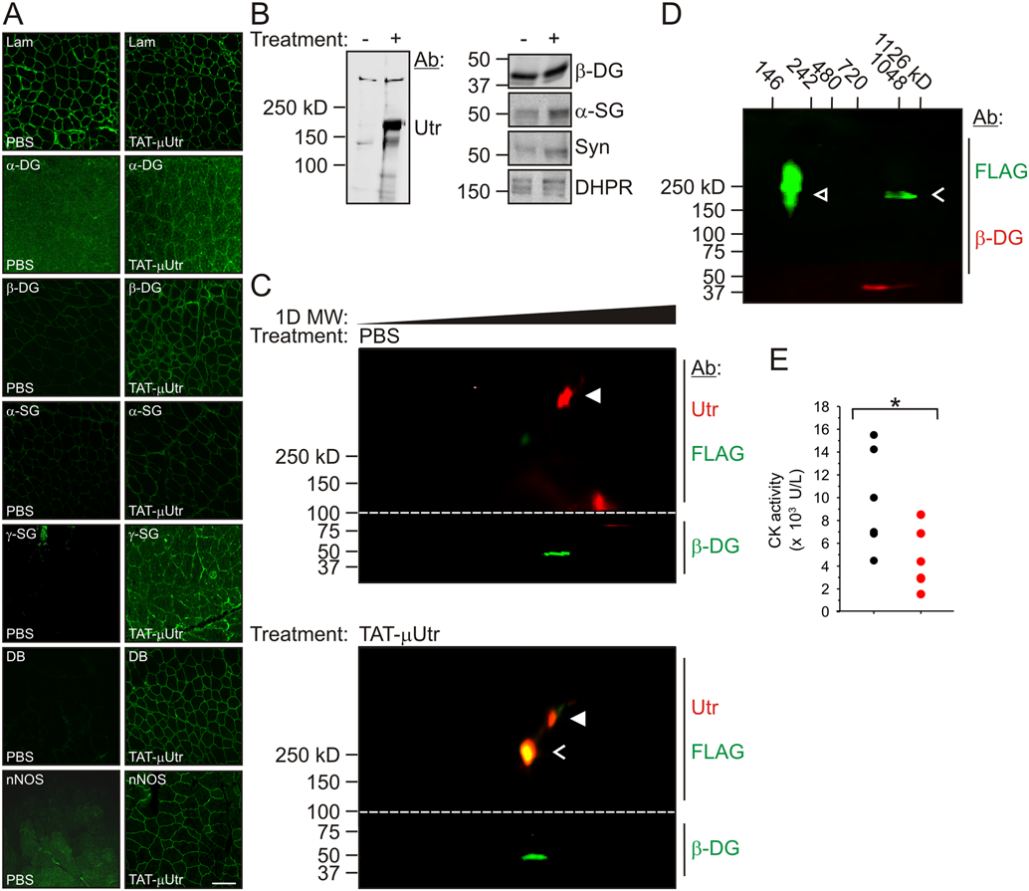
Formation of the μUGC. (A) Quadriceps muscle cryosections from mice injected with PBS and TAT-μUtr stained with antibodies to several members of the DGC (α/β-DG, α/β-dystroglycan; α/γ-SG, α/γ-sarcoglycan; DB, dystrobrevin; Lam, laminin). (B) SDS-PAGE/Western blots of WGA skeletal muscle extracts of μUGC members (DHPR, dihydropyridine receptor; Syn, syntrophin). DHPR was used as a loading control. (C) Infrared scan of Western blots after 2D BN-PAGE of skeletal muscle extracts from PBS and TAT-μUtr-injected mice after WGA affinity chromatography enrichment. 100 µg of protein was loaded per gel. Native complexes were separated by size in the first (native) dimension before complex members were resolved in the second (SDS-PAGE) dimension. Blots were cut into pieces (dotted line) to allow for β-DG and utrophin analysis of each sample and then realigned before scanning. The top half of each blot could be probed with antibodies to both utrophin (mouse mAb 8A4) and FLAG (pAb ANTI-FLAG) because secondary antibodies of different species were conjugated with distinct infrared-excitable fluores. Symbols: ▴ (filled white arrowheads), endogenous utrophin; <, complexed TAT-μUtr. (D) TAT-μUtr muscle sample analyzed as in (C) except that 0.1 µg of purified, fluorescently labeled TAT-μUtr was added to the sample prior to BN-PAGE. Symbols: <, complexed TAT-μUtr; ▵ (open white arrowheads), uncomplexed TAT-μUtr. (E) Serum levels of the muscle enzyme creatine kinase were significantly elevated in PBS (black; 11,290±920 U)- compared to TAT-μUtr (red; 5,950±1,120 U)-injected *mdx* mice. * *p* = 0.03 (Student's *t* test).

Because the loss of dystrophin expression leads to a destabilization and concomitant loss of other dystrophin–glycoprotein complex (DGC) members from the muscle cell membrane [1], we examined whether TAT-μUtr restored the DGC to the sarcolemma and improved membrane stability. Immunofluorescence analyses using antibodies raised to DGC glycoproteins α-dystroglycan, β-dystroglycan, α-sarcoglycan, and γ-sarcoglycan revealed only faint or no signal on cryosections from PBS-injected mice while each antibody probe exhibited intense staining along the periphery of muscle cells from TAT-μUtr treated mice (Figure 3A). In addition, the intracellular constituents dystrobrevin and neuronal nitric oxide synthase (nNOS) were also localized to the cell periphery of treated muscle, suggesting that administered TAT-μUtr properly interacted with endogenous dystrophin/utrophin binding partners (Figure 3A). Consistent with the immunofluorescence data in Figure 3A, TAT-μUtr and all DGC components were enriched from detergent-solubilized muscle extracts by WGA-affinity chromatography while endogenous utrophin levels remained constant (Figure 3B). These data suggest that a biochemically stable “micro” utrophin–glycoprotein complex (μUGC) formed complementary to the endogenous UGC in dystrophin-deficient muscle. To further test the stability of association between TAT-μUtr and the glycoprotein complex, we performed two-dimensional blue native polyacrylamide gel electrophoresis (2D BN-PAGE) on WGA-enriched muscle extracts from PBS- and TAT-μUtr-injected mice [20]. Western blots of samples from PBS-injected mice probed with antibodies to both utrophin and the FLAG epitope demonstrated that endogenous (FLAG-less) ∼400 kDa utrophin comigrated with β-dystroglycan in a protein complex of ∼1×10^6^ kDa (Figures 3C, S4A, and S4B), evidence that 2D BN-PAGE of WGA muscle extracts can properly resolve the endogenous UGC. Importantly, Western blots of samples from TAT-μUtr-injected mice probed for Utr and the FLAG epitope showed that both endogenous (FLAG-less) Utr and FLAG-reactive TAT-μUtr comigrated with β-DG (Figure 3C and Figure S4C). To definitively test whether the Utr/FLAG-reactive band in treated muscle extracts was aggregated TAT-μUtr that had migrated coincident with the endogenous UGC, treated muscle extracts were spiked with purified TAT-μUtr immediately prior to 2D BN-PAGE analysis. Subsequent Western blotting clearly distinguished “free” TAT-μUtr from TAT-μUtr complexed with β-DG (Figure 3D). The μUGC also mitigated the membrane stability defect of *mdx* mice, as demonstrated by a significant reduction in serum levels of the muscle enzyme creatine kinase (Figure 3E). In total, the data in Figure 3 demonstrate that TAT-μUtr successfully transduced skeletal muscle cells and formed a functional DGC-like μUGC that significantly improved membrane integrity in dystrophin-deficient muscle.

### TAT-μUtr Improves *mdx* Muscle Function

**Figure 4:**
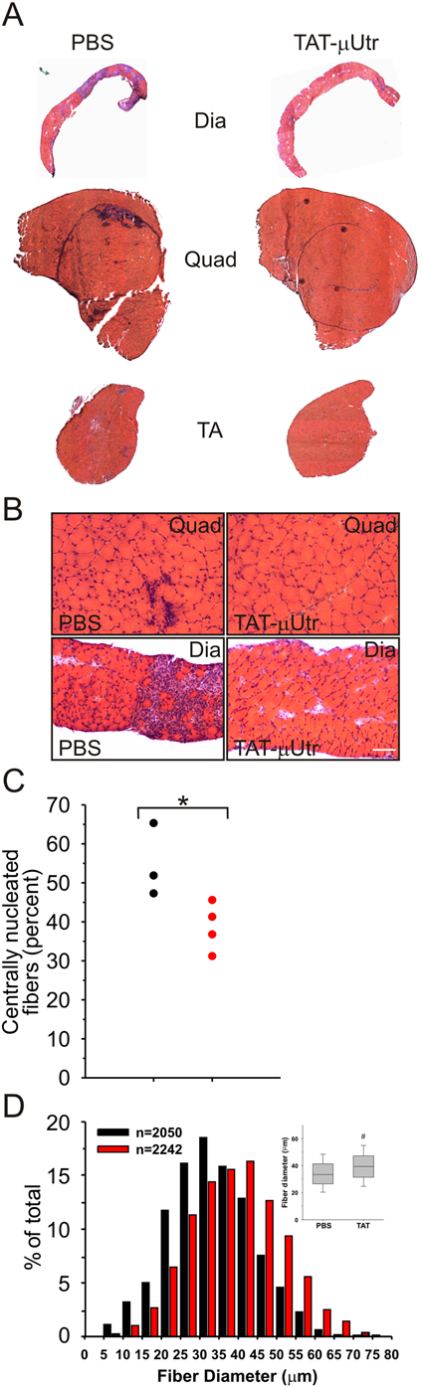
Morphology of TAT-μUtr-injected muscle. (A, B) Hematoxylin-eosin-stained whole (A) or magnified (B) diaphragm (Dia), quadriceps (Quad), and tibialis anterior (TA) muscle cryosections. Muscle from PBS-injected mice exhibited large regions of active necrosis accompanied by inflammation and small, regenerating fibers (blue-staining areas) while similar regions were dramatically reduced in TAT-μUtr treated muscle. Scale bar = 100 µm. (C) Quantification of CNFs (an index of muscle degeneration/regeneration) in quadriceps from 38-d-old PBS- (black) and TAT-μUtr (red) treated mice. Dashed line represents uninjected 38-d-old *mdx* mice. TAT-utrophin treatment led to a significant decrease in CNFs (54%±5% versus 37%±4%; PBS versus TAT; *n* = 3 PBS muscles; *n* = 4 TAT-μUtr muscles). * *p* = 0.04 (Student's *t* test). (D) Histogram: Distribution of muscle fiber diameters. Box plot: The average diameter in TAT-μUtr-treated muscles was significantly larger than PBS-injected controls (39.64±0.95 µm TAT-μUtr versus 33.94±1.15 µm PBS). # *p* = 0.02 (Student's *t* test).

Consistent with the restoration of a functional μUGC in *mdx* mice, gross histological examination of TAT-μUtr-treated muscles showed a marked improvement in the overall health of treated muscle. Hematoxylin-eosin-stained cryosections from TAT-μUtr-treated mouse muscle exhibited a general uniformity in cell size and appearance across several individual muscles, whereas sections from PBS-injected mice displayed extensive mononuclear cell infiltration and small regenerating fibers (Figure 4A and 4B). We quantified the incidence of CNFs, an index of skeletal muscle cell degeneration/regeneration, and observed significantly fewer CNFs in TAT-μUtr muscles than in PBS-injected and uninjected *mdx* controls (Figure 4C). The observed increase in fiber diameter in the TAT-μUtr-treated samples (Figure 4D) was likely due to reduced numbers of the small regenerating fibers typically found in *mdx* muscle. All three parameters indicate that TAT-μUtr administration significantly reduced muscle cell death in the absence of dystrophin.

**Figure 5:**
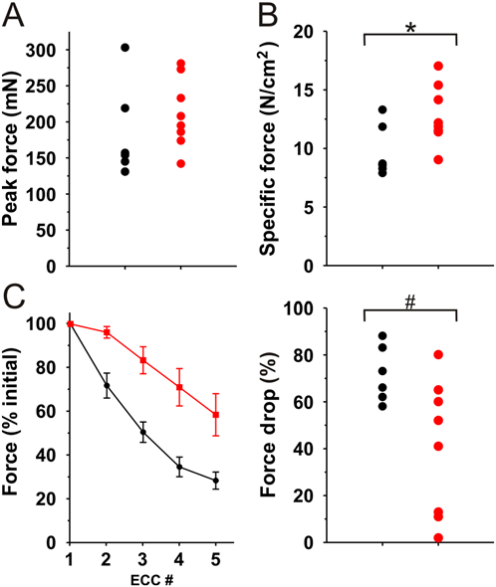
Contractile properties of TAT-μUtr treated muscle. EDL muscles dissected from mice treated with TAT-μUtr (red) exhibited *mdx*-levels of maximal tetanic force (A) but elevated levels of normalized (specific) tetanic force (B) compared to PBS-injected muscles (black). Specific force values were 9.7±1.1 N/cm^2^ (PBS) and 12.8±0.9 N/cm^2^ (TAT). * *p* = 0.03 (Student's *t* test). (C) In addition, TAT-μUtr-injected muscle was more resistant to force loss (right graph; 72±5% versus 40±8% drop; PBS versus TAT) after each of five repeated eccentric (lengthening) contractions (left graph). # *p* = 0.03 (Student's *t* test).

Dystrophin deficiency is also characterized by deficits in contractile force and a marked sensitivity to contraction-induced injury [21],[22]. Extensor digitorum longus (EDL) muscles from TAT-μUtr-treated mice showed significantly improved ex vivo functional performance (Figure 5). While *mdx* muscles typically exhibit a 25% reduction in specific force production compared to wild-type muscles [23], TAT-μUtr-treated *mdx* muscles generated comparable maximal tetanic force (Figure 5A) but ∼25% more normalized specific tetanic force (Figure 5B) than PBS-injected *mdx* control muscles. In addition, *mdx* muscle typically loses ∼75% of its force generating capacity after five lengthening (eccentric) contractions compared to ∼20% for wild-type muscle [1]. TAT-μUtr treatment significantly reduced the measured force drop by nearly half, allowing treated EDLs to maintain higher force-generating capability after each of five eccentric contractions compared to PBS-injected controls (Figure 5C). These results indicate that IP administration of TAT-μUtr significantly improved the contractile performance of dystrophin-deficient *mdx* muscle.

To test the efficacy of full-length TAT-Utr, we treated six *mdx* littermate mice as above, injecting full-length TAT-Utr at both the equivalent (1×) and varied molar ratios (0.25×, 0.5×, 2×, and 5×) to the dosage described for TAT-μUtr. Like TAT-μUtr, full-length TAT-Utr transduced all tissues examined at the 1× dosage (Figure S4B and S5A) and was properly localized to the periphery of muscle cells (Figure S5C). Similar to TAT-μUtr, full-length TAT-Utr significantly reduced muscle degeneration/regeneration (Figure S6A–S6D) and improved membrane stability (Figure S6E). However, muscle contractile performance was not significantly improved by full-length TAT-Utr even when tested at 5-fold higher dosages (Figure S6F). We conclude that the enhanced protein transduction gained by reducing the molecular weight of utrophin overcomes any loss of TAT-μUtr's biological function and propose TAT-μUtr as a potential direct protein replacement therapy for dystrophinopathies.

## Discussion

Our experiments are, to our knowledge, the first to demonstrate the feasibility and efficacy of direct protein replacement to combat the effects of dystrophin deficiency in *mdx* mice, an established model of Duchenne muscular dystrophy in humans. IP injections of the cell-penetrating TAT-μUtr restored proper membrane targeting of dystrophin protein complex members, stabilized muscle membrane integrity, attenuated histological hallmarks of dystrophy, and conferred functional benefits to treated muscle.

The initial studies in which dystrophin deficiency was corrected by a reintroduction of either dystrophin [24] or utrophin expression [10] caused widespread excitement that an effective treatment would soon be realized. Despite consistent progress over the intervening years, obstacles to gene-, oligonucleotide-, or small molecule-based approaches to up-regulate the relevant proteins still exist and have thus far prevented effective therapeutic intervention in human patients. Our development of TAT-μUtr and demonstration of its beneficial effects on dystrophic muscle—all without affecting endogenous utrophin levels or function—introduces a novel therapeutic approach that we predict would work complementarily and synergistically with other strategies, in particular those aimed at up-regulating endogenous utrophin expression [25],[26]. The beneficial effects of combining such therapies should theoretically be additive with the prediction that the cumulative increase in utrophin levels would be sufficient to substantially prevent or delay the phenotypes associated with dystrophin deficiency.

Our proof-of-concept results offer the first evidence, as far as we know, that an exogenous protein replacement-based therapy holds promise for the treatment of dystrophinopathies. Besides the obvious example of Duchenne muscular dystrophy, we are intrigued by the possibility that TAT-μUtr may also be able to compensate for the loss of dystrophin in genetic or acute forms of cardiomyopathy [27],[28].

## Supporting Information

Figure S1 **TAT-Utr expression and actin binding.** (A) Protein schematic and Coomassie brilliant blue gel of purified protein. (B) Actin binding properties of TAT-Utr and TAT-μUtr. Actin cosedimentation assays were performed as previously described [1], except the concentration of utrophin was held constant while the concentration of actin was varied. The TAT domain did not interfere with actin binding in the full-length TAT-Utr construct (0.22 versus 0.11 µM), while deletion of spectrin-like repeats 4–21 caused a 5- to 10-fold reduction in actin binding affinity for TAT-μUtr (0.22 versus 1.14 µM). *n*≥3 for each protein.
(0.68 MB TIF)

Figure S2 **Tissue transduction of TAT-μUtr.** (A) Li-Cor Odyssey-scanned SDS-PAGE gel (left) and subsequent Western blot (right) of lung, brain, liver, kidney, spleen, heart, triceps, quadriceps, and tibialis anterior SDS protein extracts from mice that were injected with fluorescently labeled TAT-μUtr. The gel was not probed with antibody; signal only corresponds to the labeled protein. The Western blot membrane was cut into two pieces and probed with FLAG antibody (top) and actin antibody (bottom). (B) Li-Cor Odyssey-scanned tissue cryosections from mice in (A). Green signal indicates fluorescently labeled, TAT-μUtr-transduced entire muscle tissues, although the periphery of the quadriceps was more strongly transduced. (C, D) Li-Cor Odyssey-scanned whole organs and tissues from mice injected with labeled TAT-μUtr (two mice), PBS, and labeled full-length TAT-Utr as in (A) show systemic uptake of the labeled TAT-μUtr. Red channel indicates autofluorescence in the 700 nm channel. Muscles magnified in (D) are the same samples depicted in (C). (E) Quantification of fluorescence intensity of organs and tissues in (C). Note that organs and tissues lining or in the peritoneal space exhibited the strongest signal, although all organs from TAT-μUtr-injected mice emitted fluorescence compared to PBS-injected mice. All sample intensities were normalized to PBS samples to obtain relative values. (F) Western blot analysis of quadriceps and liver SDS extracts from TAT-μUtr (μ), TAT-Utr (FL), and PBS- (−) treated mice demonstrated higher levels of TAT-μUtr than TAT-Utr in the respective tissues.
(0.77 MB TIF)

Figure S3 **Full-length TAT-Utr stability.** (A) Infrared in vivo scanning of *mdx* littermate mice after a single IP injection of fluorescently labeled full-length TAT-Utr. Mice were scanned 3, 24, 48, and 72 h postinjection, with one mouse killed at each time point for tissue analysis. Green fluorescence corresponds to full-length TAT-Utr while red signal is tissue autofluorescence. (B) Quantification of the whole-body fluorescence decay over time in (A).
(0.35 MB TIF)

Figure S4 **2D BN-PAGE.** (A) Li-Cor Odyssey-scanned blue native gel loaded with 100 µg of WGA muscle extract from a mouse injected with fluorescently labeled TAT-μUtr showed that the 167 kDa TAT-μUtr migrated with a complex of ∼1×10^6^ kDa. The gel was not probed with antibody; green signal only corresponds to the labeled protein. (B, C) Western blot analysis of blue native gels as in (A) resolved by SDS-PAGE to allow for the identification of complex members. Blots were cut into two separate pieces to allow individual samples to be probed with antibodies to utrophin (mAb 8A4), FLAG (pAb ANTI-FLAG), and dystroglycan (mAb b-DG) simultaneously. Only endogenous full-length utrophin comigrated with dystroglycan in PBS-injected mice (B) while both endogenous full-length and TAT-μUtr comigrated with dystroglycan in TAT-μUtr-treated mice (C).
(1.04 MB TIF)

Figure S5 **Full-length TAT-Utr transduction.** (A) Western blot analysis of SDS-extracts from 38-d-old *mdx* mice after sham PBS-treatment (−) or 1× TAT-Utr treatment (+) probed with a utrophin-specific polyclonal antibody [29]. A similar increase in utrophin levels was observed in all tissues examined. (B) Western blot analysis of gastrocnemius, quadriceps, or whole skeletal muscle SDS-extracts probed with an antibody specific to the FLAG epitope of TAT-Utr demonstrated the presence of TAT-Utr in each muscle tissue. (C) Immunofluorescence analysis on 10 µm thick cryosections from PBS- and TAT-Utr-treated *mdx* mice using antibodies specific to utrophin (NCL-DRP2) or the HA-epitope (HA.11) on TAT-Utr. Scale bar = 100 µm.
(0.47 MB TIF)

Figure S6 **Full-length TAT-Utr improves several dystrophic parameters in** ***mdx*** **mice.** Hematoxylin-eosin-stained whole (A) or magnified (B) quadriceps or tibialis anterior muscle cryosections. Muscle from PBS-injected mice exhibited large regions of active necrosis accompanied by inflammation and small, regenerating fibers (blue-staining areas), while similar regions were dramatically reduced in 1× TAT-Utr treated muscle. Scale bar = 100 µm. (C) Quantification of CNFs (an index of muscle degeneration/regeneration) in tibialis anterior and quadriceps from 38-d-old PBS- (black bars) and 0.25× (gray), 0.5× (yellow), 1× (blue), 2× (orange), and 5× (white) TAT-Utr treated mice. Dashed line represents un-injected 38-d-old *mdx* mice. TAT-Utr treatment led to a 40% decrease in CNFs (*n* = 5 muscles/group). (*) denotes *p* = 0.03. (D) Histogram: Distribution of muscle fiber diameters demonstrated a larger proportion of small fibers in PBS-injected control mice. Box plot: The average diameter in 1× TAT-Utr treated muscle was significantly larger (37.94±1.14 µm for 1× TAT-Utr versus 34.94±1.30 µm for PBS, * *p* = 0.03). (E) Serum activity levels of the muscle enzyme creatine kinase were reduced 50% in 38-d-old TAT-Utr treated mice compared to PBS-injected controls. * *p* = 0.01. (F) Maximal tetanic force generation, (G) specific force generation, or (H) susceptibility to contraction-induced injury was not improved by any dosage of full-length TAT-Utr.
(2.51 MB TIF)

Text S1 **Supplementary methods.**
(0.03 MB DOC)

## Abbreviations

BN-PAGE: blue native PAGE
CNF: centrally nucleated fiber
β-DG: β-dystroglycan
DGC: dystrophin–glycoprotein complex
DMD: Duchenne muscular dystrophy
EDL: extensor digitorum longus
IP: intraperitoneal
nNOS: neuronal nitric oxide synthase
PTD: protein transduction domain
UGC: utrophin–glycoprotein complex
μUGC: “micro” utrophin–glycoprotein complex
Utr: utrophin
μUtr: ΔR4-21 “micro” utrophin
WGA: wheat germ agglutinin
