# Supplementary figures and images for: Functional Substitution by TAT-Utrophin in Dystrophin-Deficient Mice
Source: PLoS Med. 2009 May 26;6(5):e1000083. doi: 10.1371/journal.pmed.1000083 (PMC2680620; doi:10.1371/journal.pmed.1000083)

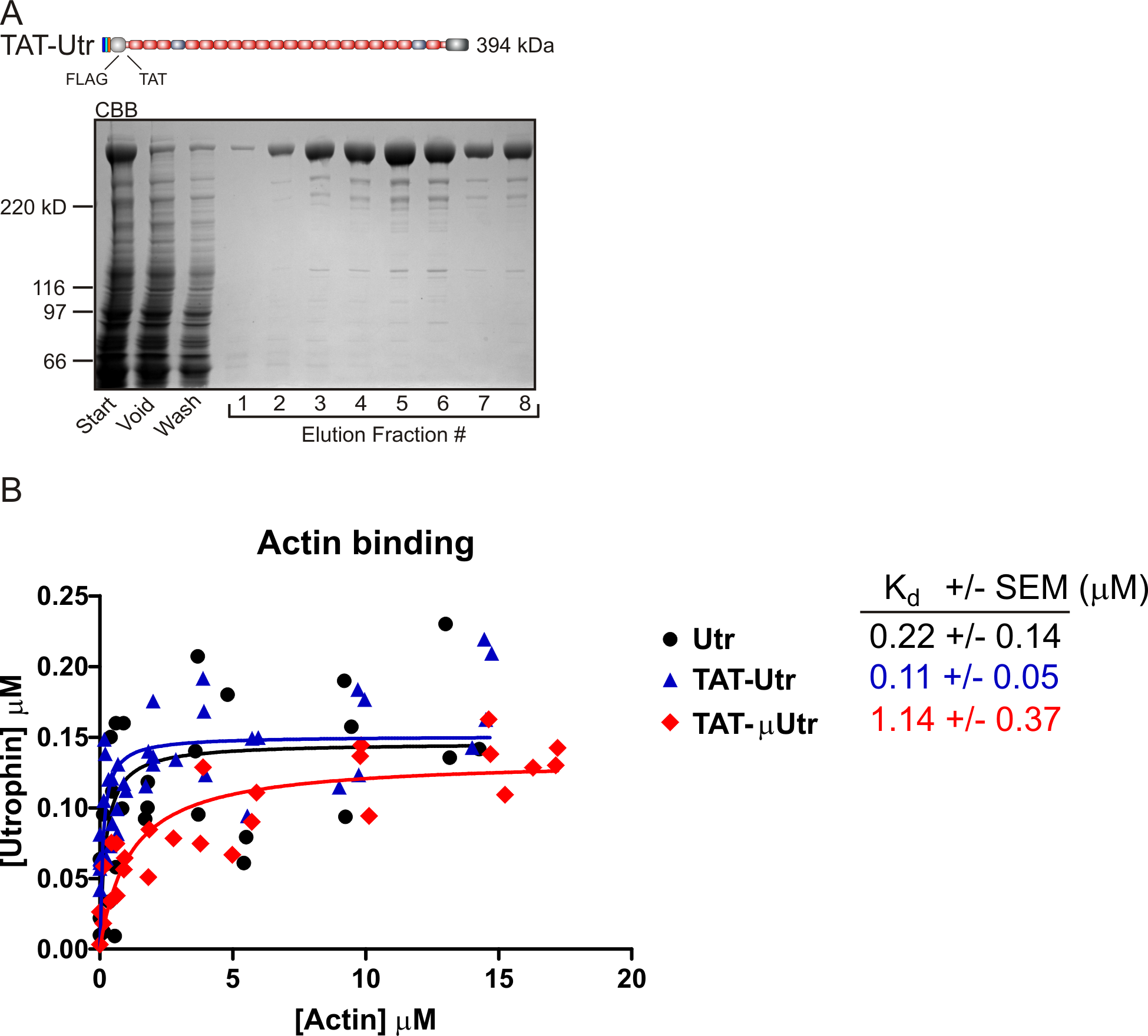

Supplement: Figure S1 — TAT-Utr expression and actin binding. (A) Protein schematic and Coomassie brilliant blue gel of purified protein. (B) Actin binding properties of TAT-Utr and TAT-μUtr. Actin cosedimentation assays were performed as previously described [1], except the concentration of utrophin was held constant while the concentration of actin was varied. The TAT domain did not interfere with actin binding in the full-length TAT-Utr construct (0.22 versus 0.11 µM), while deletion of spectrin-like repeats 4–21 caused a 5- to 10-fold reduction in actin binding affinity for TAT-μUtr (0.22 versus 1.14 µM). n≥3 for each protein. (0.68 MB TIF) [file pmed.1000083.s001.tif]

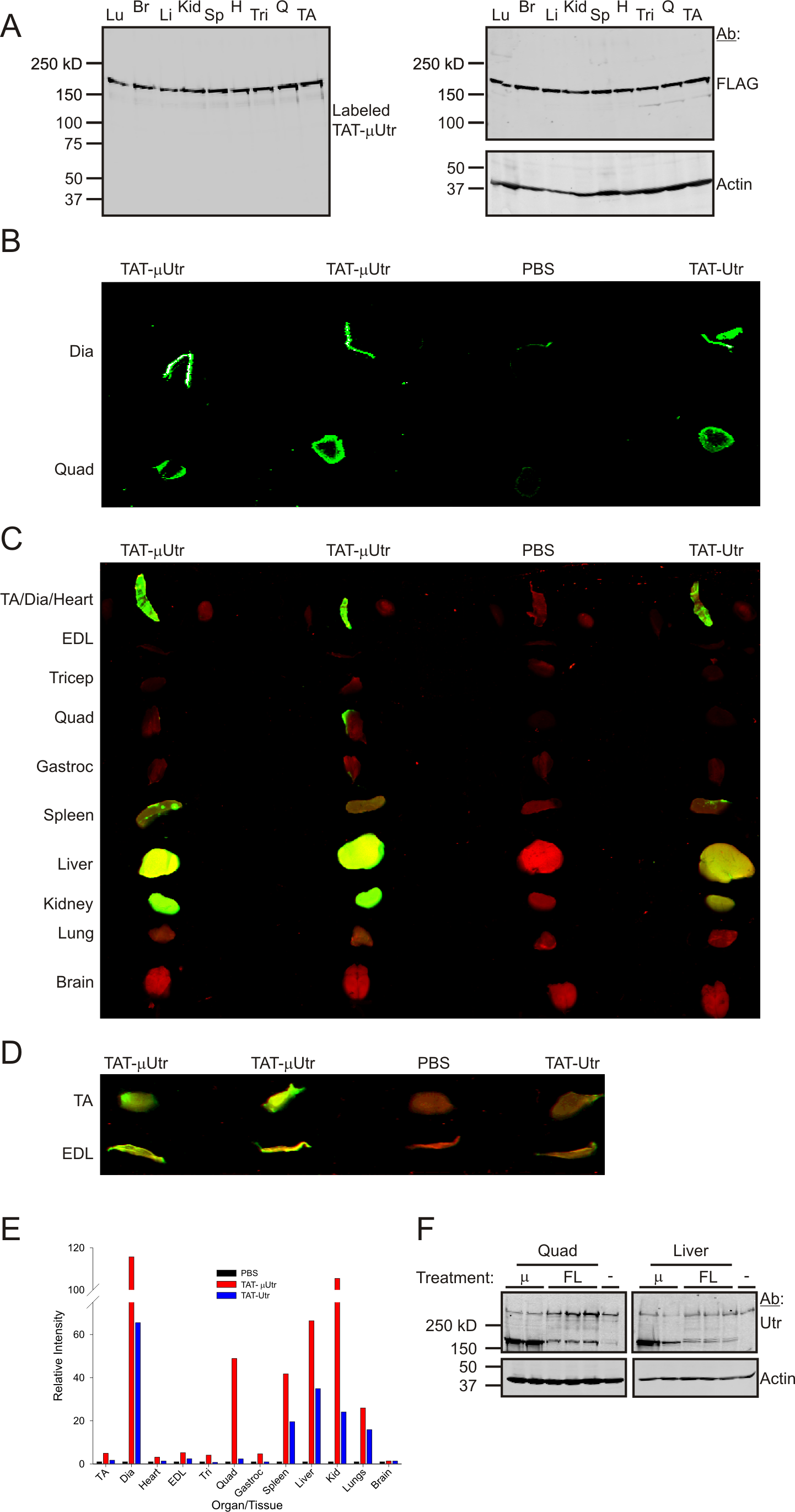

Supplement: Figure S2 — Tissue transduction of TAT-μUtr. (A) Li-Cor Odyssey-scanned SDS-PAGE gel (left) and subsequent Western blot (right) of lung, brain, liver, kidney, spleen, heart, triceps, quadriceps, and tibialis anterior SDS protein extracts from mice that were injected with fluorescently labeled TAT-μUtr. The gel was not probed with antibody; signal only corresponds to the labeled protein. The Western blot membrane was cut into two pieces and probed with FLAG antibody (top) and actin antibody (bottom). (B) Li-Cor Odyssey-scanned tissue cryosections from mice in (A). Green signal indicates fluorescently labeled, TAT-μUtr-transduced entire muscle tissues, although the periphery of the quadriceps was more strongly transduced. (C, D) Li-Cor Odyssey-scanned whole organs and tissues from mice injected with labeled TAT-μUtr (two mice), PBS, and labeled full-length TAT-Utr as in (A) show systemic uptake of the labeled TAT-μUtr. Red channel indicates autofluorescence in the 700 nm channel. Muscles magnified in (D) are the same samples depicted in (C). (E) Quantification of fluorescence intensity of organs and tissues in (C). Note that organs and tissues lining or in the peritoneal space exhibited the strongest signal, although all organs from TAT-μUtr-injected mice emitted fluorescence compared to PBS-injected mice. All sample intensities were normalized to PBS samples to obtain relative values. (F) Western blot analysis of quadriceps and liver SDS extracts from TAT-μUtr (μ), TAT-Utr (FL), and PBS- (−) treated mice demonstrated higher levels of TAT-μUtr than TAT-Utr in the respective tissues. (0.77 MB TIF) [file pmed.1000083.s002.tif]

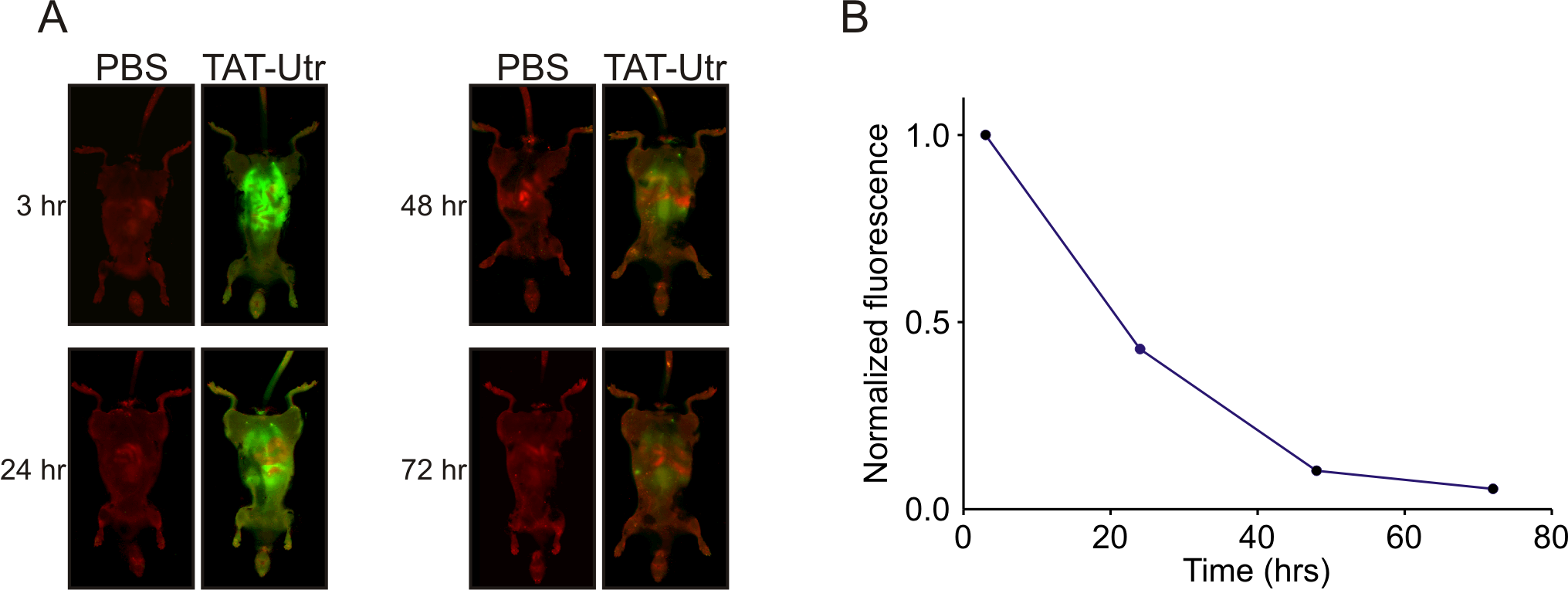

Supplement: Figure S3 — Full-length TAT-Utr stability. (A) Infrared in vivo scanning of mdx littermate mice after a single IP injection of fluorescently labeled full-length TAT-Utr. Mice were scanned 3, 24, 48, and 72 h postinjection, with one mouse killed at each time point for tissue analysis. Green fluorescence corresponds to full-length TAT-Utr while red signal is tissue autofluorescence. (B) Quantification of the whole-body fluorescence decay over time in (A). (0.35 MB TIF) [file pmed.1000083.s003.tif]

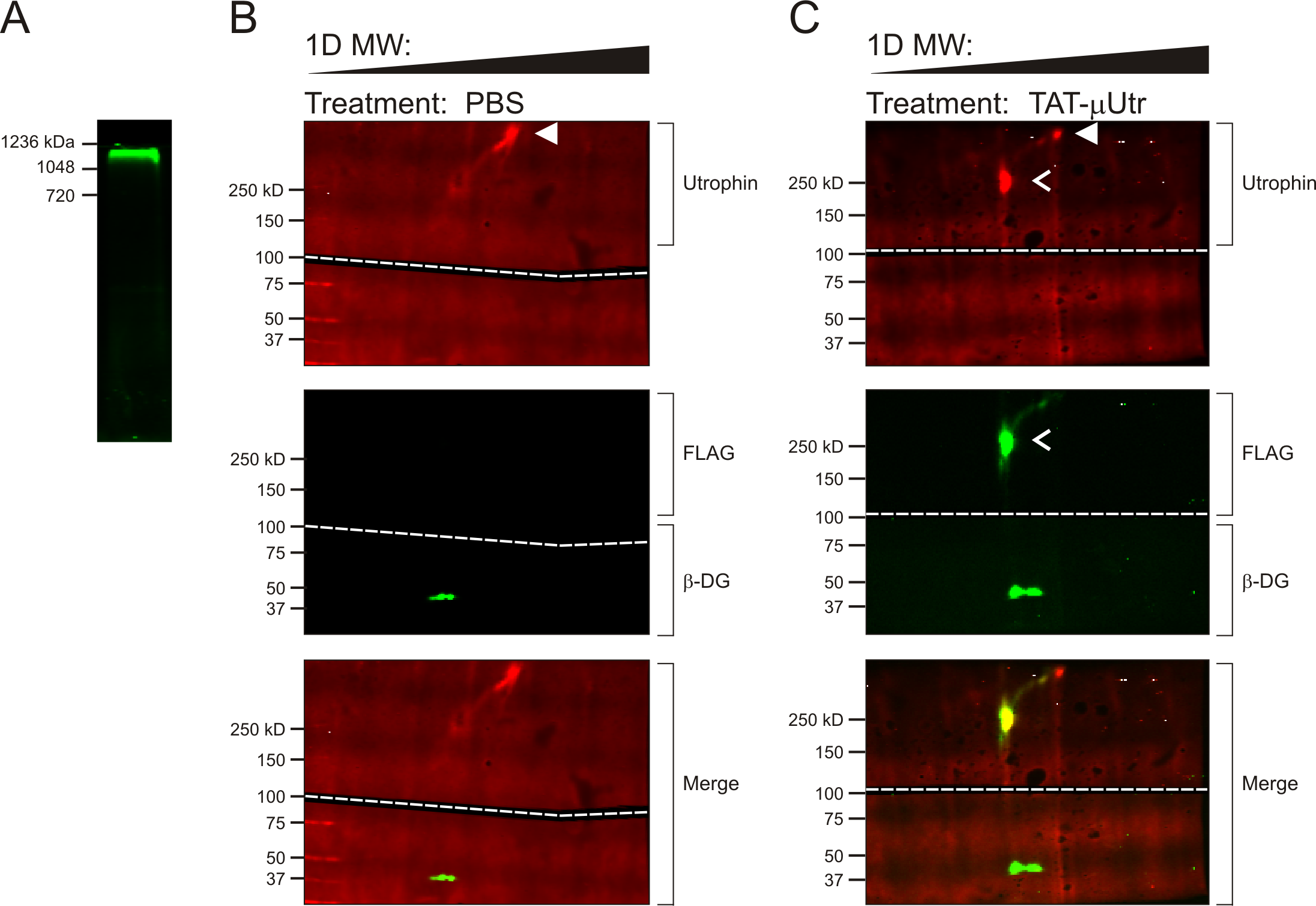

Supplement: Figure S4 — 2D BN-PAGE. (A) Li-Cor Odyssey-scanned blue native gel loaded with 100 µg of WGA muscle extract from a mouse injected with fluorescently labeled TAT-μUtr showed that the 167 kDa TAT-μUtr migrated with a complex of ∼1×10^6^ kDa. The gel was not probed with antibody; green signal only corresponds to the labeled protein. (B, C) Western blot analysis of blue native gels as in (A) resolved by SDS-PAGE to allow for the identification of complex members. Blots were cut into two separate pieces to allow individual samples to be probed with antibodies to utrophin (mAb 8A4), FLAG (pAb ANTI-FLAG), and dystroglycan (mAb b-DG) simultaneously. Only endogenous full-length utrophin comigrated with dystroglycan in PBS-injected mice (B) while both endogenous full-length and TAT-μUtr comigrated with dystroglycan in TAT-μUtr-treated mice (C). (1.04 MB TIF) [file pmed.1000083.s004.tif]

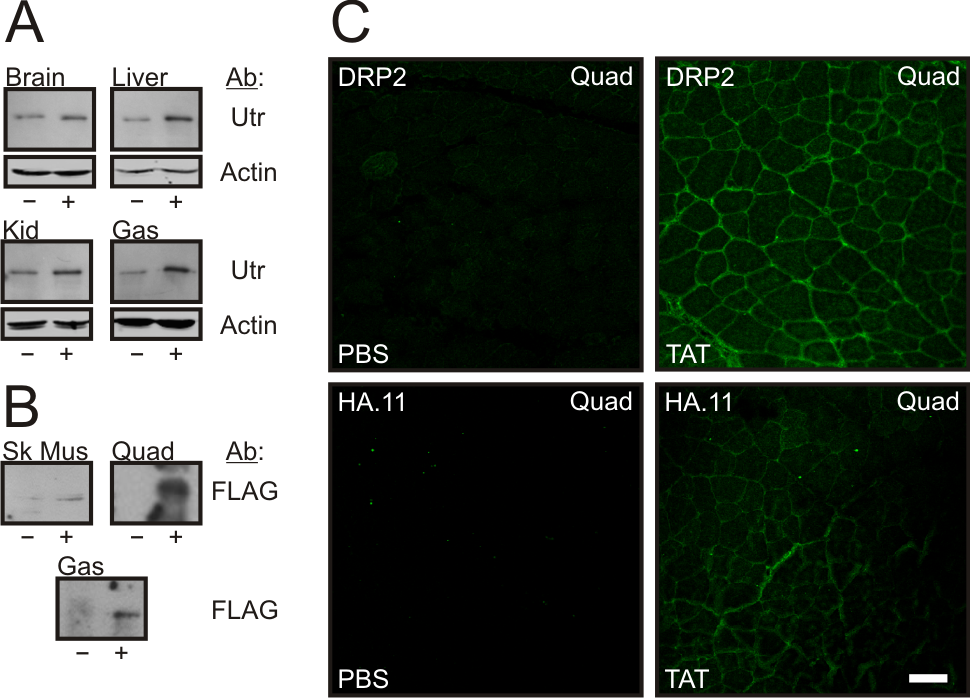

Supplement: Figure S5 — Full-length TAT-Utr transduction. (A) Western blot analysis of SDS-extracts from 38-d-old mdx mice after sham PBS-treatment (−) or 1× TAT-Utr treatment (+) probed with a utrophin-specific polyclonal antibody [29]. A similar increase in utrophin levels was observed in all tissues examined. (B) Western blot analysis of gastrocnemius, quadriceps, or whole skeletal muscle SDS-extracts probed with an antibody specific to the FLAG epitope of TAT-Utr demonstrated the presence of TAT-Utr in each muscle tissue. (C) Immunofluorescence analysis on 10 µm thick cryosections from PBS- and TAT-Utr-treated mdx mice using antibodies specific to utrophin (NCL-DRP2) or the HA-epitope (HA.11) on TAT-Utr. Scale bar = 100 µm. (0.47 MB TIF) [file pmed.1000083.s005.tif]

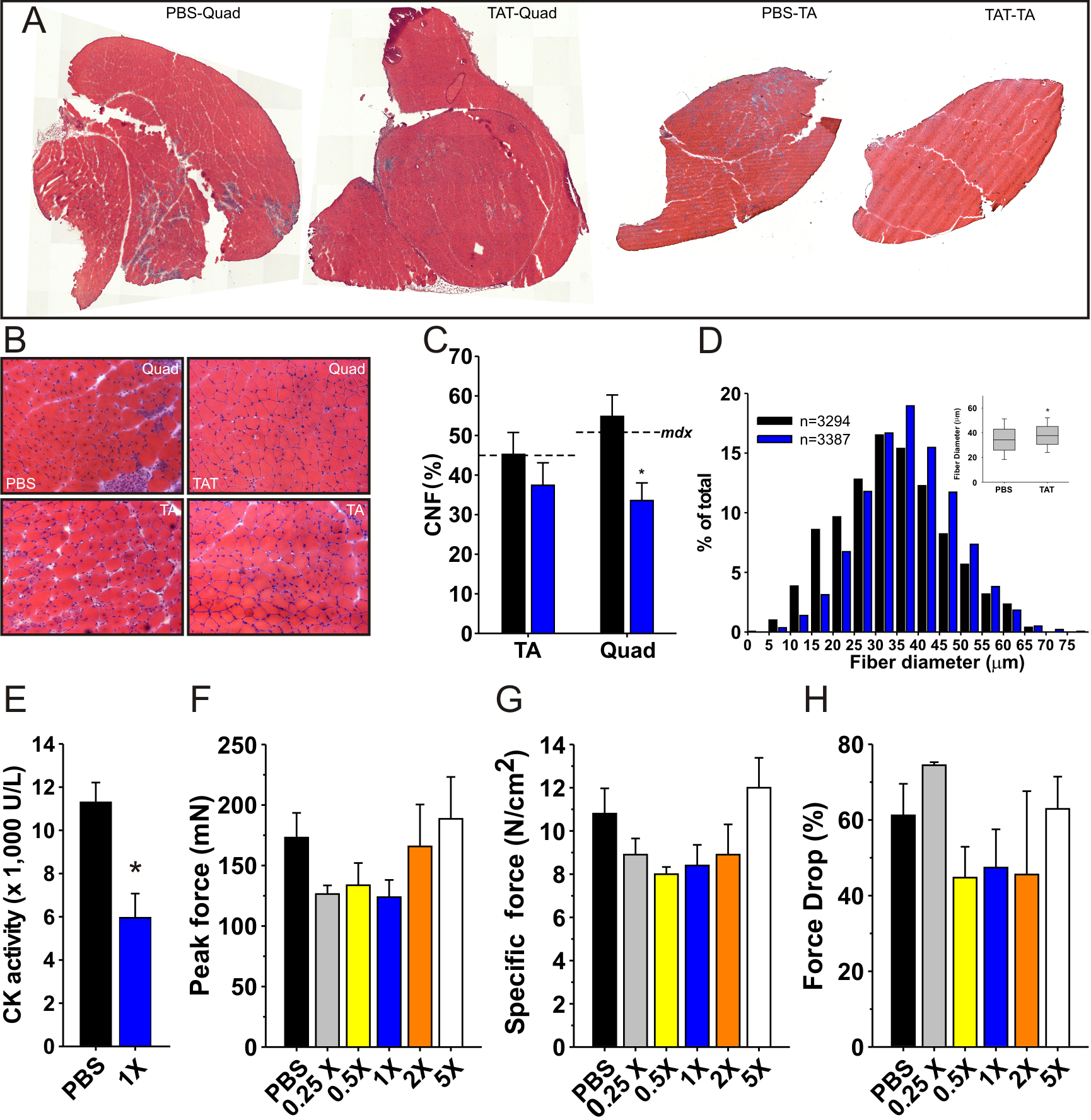

Supplement: Figure S6 — Full-length TAT-Utr improves several dystrophic parameters in mdx mice. Hematoxylin-eosin-stained whole (A) or magnified (B) quadriceps or tibialis anterior muscle cryosections. Muscle from PBS-injected mice exhibited large regions of active necrosis accompanied by inflammation and small, regenerating fibers (blue-staining areas), while similar regions were dramatically reduced in 1× TAT-Utr treated muscle. Scale bar = 100 µm. (C) Quantification of CNFs (an index of muscle degeneration/regeneration) in tibialis anterior and quadriceps from 38-d-old PBS- (black bars) and 0.25× (gray), 0.5× (yellow), 1× (blue), 2× (orange), and 5× (white) TAT-Utr treated mice. Dashed line represents un-injected 38-d-old mdx mice. TAT-Utr treatment led to a 40% decrease in CNFs (n = 5 muscles/group). (*) denotes p = 0.03. (D) Histogram: Distribution of muscle fiber diameters demonstrated a larger proportion of small fibers in PBS-injected control mice. Box plot: The average diameter in 1× TAT-Utr treated muscle was significantly larger (37.94±1.14 µm for 1× TAT-Utr versus 34.94±1.30 µm for PBS, * p = 0.03). (E) Serum activity levels of the muscle enzyme creatine kinase were reduced 50% in 38-d-old TAT-Utr treated mice compared to PBS-injected controls. * p = 0.01. (F) Maximal tetanic force generation, (G) specific force generation, or (H) susceptibility to contraction-induced injury was not improved by any dosage of full-length TAT-Utr. (2.51 MB TIF) [file pmed.1000083.s006.tif]
