# Supplementary material for: Functional Substitution by TAT-Utrophin in Dystrophin-Deficient Mice
Source: PLoS Med. 2009 May 26;6(5):e1000083. doi: 10.1371/journal.pmed.1000083 (PMC2680620; doi:10.1371/journal.pmed.1000083)
Supplement: Text S1 — Supplementary methods. (0.03 MB DOC) [file pmed.1000083.s007.doc]

**SUPPLEMENTARY INFORMATION**

**METHODS**

*TAT-μUtr construction* - TAT-μUtr cDNA was produced using recombinant PCR. In the first step, the two recombination fragments were amplified separately. The 5’ fragment was generated with two primers, one of which contained an overhanging sequence complementary to the other recombination fragment: *AGGTCTCGGAGTTTCTCTAA*GTCCACGTGAATCTGTCTCTTCTTT. This primer was complementary to one of the primers used for the 3’ fragment: *TTAGAGAAACTCCGAGACCT*GCAGG (overlap in *italics*). The 5’ and 3’ fragments were combined in a subsequent PCR, in which one strand of the 5’ fragment acted as a megaprimer. No additional primers were necessary in the reaction. After 5 cycles (95°C for 15 sec, 45°C for 15 sec, 72°C for 6 min), outer primers (GTTCTAGTGGTTGGCTACGTATACTCC and GTCGTATTCAGCTCTAAGAGATCCAGAC) were added and PCR was completed in 24 cycles at regular annealing temperature (95°C for 15 sec, 53°C for 15 sec, 72°C for 6 min). The recombined PCR product was then inserted into a pFastBac1 (Invitrogen) vector containing the 3’-end of full-length TAT-Utrophin by restriction digest with NotI and BstBI. *Pfu* Ultra polymerase was used for all PCR, and all constructs were confirmed by DNA sequence analysis.

*Actin binding* - Recombinant FLAG-tagged wild-type full-length utrophin, full-length TAT-Utr, and TAT-μUtr binding to muscle actin was measured by high-speed cosedimentation analysis largely as described previously [1]. However, utrophin protein concentrations were held constant at 0.5 μM and incubated with actin concentrations varying from 0.2 to 18 μM.

*Contractile properties* - Mice were anesthetized with sodium pentobarbital (100 mg/kg BW) before the EDL muscles were dissected and mounted to a dual-mode muscle lever system (300B-LR; Aurora Scientific Inc., Aurora, ON, Canada) with 6-0 suture in a 0.38-ml bath assembly filled with Krebs–Ringer bicarbonate buffer that was maintained at 25°C and perfused with 95% O_2_. Muscles were adjusted to their anatomic optimal length (L_o_) based on resting tension of 0.4 g and then muscle length was measured [2]. Muscles remained quiescent in the bath for 10 min. Two maximal isometric tetanic contractions (P_o_) were performed by stimulating muscles for 400 ms at 180 Hz and 150 V (Grass S48 stimulator delivered through a SIU5D stimulus isolation unit; Grass Telefactor, Warwick, RI) with a rest period of 2 min between contractions. The higher of the two P_o_ measurements was used to calculate specific force. Specific force was calculated by normalizing P_o_ to muscle cross-sectional area [muscle weight divided by the product of muscle density (1.06 g/ml) and fiber length]. Fiber length was assumed to be 44% of EDL muscle length. Two minutes later, an injury protocol consisting of 5 eccentric contractions was performed. For these contractions, muscles were passively shortened from L_o_ to 0.95 L_o_ over 3 s, stimulated tetanically for 200 ms as the muscle lengthened to 1.05 L_o_ at 0.5 L_o_/s, and then passively returned to L_o_. Each eccentric contraction was separated by 3 min of rest. Force drop was calculated as (ECC1-ECC5)/ECC1. Contractility and injury protocols were performed on EDL muscles from both the right and left legs and averaged together as a single data point for each mouse. Data were compared using a student’s t-test (TAT-μUtr) or ANOVA (full-length TAT-Utr).

REFERENCES

1. Rybakova IN, Patel JR, Davies KE, Yurchenco PD, Ervasti JM (2002) Utrophin binds laterally along actin filaments and can couple costameric actin with the sarcolemma when overexpressed in dystrophin-deficient muscle. Mol Biol Cell 13: 1512-1521.

2. Petrof BJ, Shrager JB, Stedman HH, Kelly AM, Sweeney HL (1993) Dystrophin protects the sarcolemma from stresses developed during muscle contraction. Proc Natl Acad Sci USA 90: 3710-3714.
